# Potentially inappropriate medications used by the elderly: prevalence and risk factors in Brazilian care homes

**DOI:** 10.1186/1471-2318-13-52

**Published:** 2013-05-30

**Authors:** Thaís Jaqueline Vieira de Lima, Cléa Adas Saliba Garbin, Artênio José Ísper Garbin, Dóris Hissako Sumida, Orlando Saliba

**Affiliations:** 1 Department of Child and Social Dentistry, Dental School of Araçatuba, Univ Estadual Paulista-UNESP (São Paulo State University), 1193 José Bonifácio Street, Araçatuba, SP 16015-050, Brazil; 2 Department of Basic Sciences, Dental School of Araçatuba, Univ Estadual Paulista-UNESP (São Paulo State University), Araçatuba, SP, Brazil

**Keywords:** Drug utilization, Aged, Inappropriate prescribing

## Abstract

**Background:**

The use of potentially inappropriate medications (PIM) among the elderly is a serious public health problem because it is intrinsically linked to increased morbidity and mortality, causing high costs to public health systems. This study’s objective was to verify the prevalence of and the factors associated with the use of PIMs by elderly Brazilians in institutional settings.

**Methods:**

We performed a transversal study, by consulting the case files of elderly people living in Long Term Care for the Elderly (LTC) in towns in the State of São Paulo, Brazil, as well as structured interviews with the nurses responsible for them.

We identified PIMs using the list of recently updated Beers criteria developed by a group of specialists from the American Geriatrics Society (AGS), who reviewed the criteria based on studies with high scientific evidence levels. We defined the factors studied to evaluate the association with PIM use prior to the statistical analyses, which were the chi-square test and multiple logistic regression.

**Results:**

Among the elderly who used drugs daily, 82.6% were taking at least one PIM, with antipsychotics (26.5%) and analgesics (15.1%) being the most commonly used. Out of all the medications used, 32.4% were PIMs, with 29.7% of these being PIMs that the elderly should avoid independent of their condition, 1.1% being inappropriate medication for older adults with certain illnesses or syndromes, and 1.6% being medications that older adults should use with caution. In the multivariate analysis, the factors associated with PIM use were: polypharmacy (p = 0.0187), cerebrovascular disease (p = 0.0036), psychiatric disorders (p < 0.0001) and dependency (p = 0.0404).

**Conclusions:**

The results of this study showed a high prevalence of PIM use in institutionalized elderly Brazilian patients. and the associated factors were polypharmacy, psychiatric disorders, cerebrovascular diseases and dependency.

## Background

The elderly’s use of inappropriate medications is a serious public health problem because it is intimately related to adverse drug reactions [1,2], and these, in turn, lead to an exceptional increase in indices of morbidity and mortality, generating wasted health resources [3], especially among the very old [4].

The use of potentially inappropriate medications (PIMs) has been studied in different health care scenarios, and despite the wide range of information on the subject, health professionals continue to prescribe many PIMs to the elderly. The use of PIMs among residents of long-term care for the elderly (LTC)—which, according to Morais [5], consist of groups of elderly people with particularly high uses of medications differs according to the countries where the studies have been performed. In Japan, Niwata et al. [6] found a PIM use rate of 21.1% in LTC, which included not only nursing homes, but also other institutions, such as hospitals and elderly care centers. In Malaysia, the PIM use rate among the elderly residents in an LTC was 32.7% [1].

In a review of studies performed in LTCs in Europe and the United States, Gallagher et al. [7] found a prevalence of 40%.

After an extensive review of the existing literature, we found only one study performed in a Brazilian LTC by Castellar et al. [8], who found a PIM use prevalence of 46.2%.

Holistic therapeutic care, including pharmaceutical care, is an area of interest for the Brazilian Public Health System (Sistema Único de Saúde [SUS]), which plays an essential role in guaranteeing access to medication for the whole population [9]. However, according to Gomes and Caldas [10], medical professionals in Brazil lack knowledge about prescribing PIMs to the elderly. In addition, the fact that the SUS does not have the most adequate medications for the elderly regularly available increases the risk of inappropriate medications being given to this group.

The Beers Criteria are used to classify and describe the risks related to these medications [11-13]. For 20 years, the Beers Criteria list has been the most widely consulted list for evaluating the prescription of medication to the elderly [14,15].

However, these criteria must be updated regularly to reflect newly launched medications and the publication of new evidence related to the use of these medications. As a result, a multidisciplinary panel of specialists (American Geriatrics Society [AGS]) after a rigorous systematic review, recently updated and expanded the criteria [16].

The medications included in the new list are subdivided into three main categories: 34 medications that are potentially inappropriate because they have a high risk of adverse reactions or are of limited effectiveness in the elderly; 14 medications that are potentially inappropriate for elderly people with certain pathologies and/or syndromes since they can exacerbate the pre-existing disease/syndrome; 14 medications that should be used with caution by the elderly. These medications can be associated with greater risk than benefit in the elderly [16]. In light of this situation, the main objectives of this study were to verify the prevalence of prescribing inappropriate medications to the elderly by using the updated Beers Criteria and to determine what factors are associated with inappropriate prescriptions in elderly institutionalized Brazilians.

## Methods

### Study design

We performed a transversal study by consulting the files of elderly residents of care homes, with an age of ≥60 years and through structured interviews with the nurses responsible for the elderly residents in these institutions during the period from December 2011 to May 2012.

We applied an age limit of 60 years for the sample participants. This age limit is justified because in developing countries, such as Brazil, the World Health Organization (WHO) [17] considers people to be elderly when they are 60 years or older. This threshold differs from that in developed countries, where the age limit is 65 years or above. Moreover, in Brazil, certain legal measures describe the elderly population based on this age range [18,19].

We performed data collection in four towns in the state of São Paulo, Brazil. Three of these towns were large (population between 100,000 and 900,000 inhabitants) and one was medium sized (population between 50,001 and 100,000 inhabitants) [20]. The selection of towns was by convenience. To obtain a more homogenous sample, the study population included all of the elderly people living in all six of the LTCs in these four towns.

We excluded people who had not yet reached 60 years of age during the data collection period, those who did not fully reside at the institution, and those who died during this period.

The six institutions housed elderly people with the same socio-economic characteristics. The majority of the population was illiterate, had a low educational level, and had retired on a low income.

Data was obtained by documentary analysis of the medical records of the elderly residents, complemented by interviews with the nurses responsible for each LTC. For the interviews, we used a previously tested structured questionnaire with open and closed questions, and we recorded the following data: social characteristics (age, household income, educational level) and questions related to health, such as hospital admissions during the year, pathologies, symptoms or syndromes present, as well as a diary of the medications prescribed (name of medication, indication for medication, and symptoms after taking the medication). We also recorded data that evaluated the dependency level, using the Katz Index [21], which was applied to all the medical records.

During the month prior to data collection, all the medical drugs prescribed were collected, during which all standard information contained in the records was registered and the daily prescription charts or prescriptions for each resident reviewed, complemented when necessary by information from the packaging and leaflets provided. All industrial medical drugs and master formulas were included in the database and analyzed, except for those whose composition could not be clearly determined (homeopathics, phytotherapies and teas). The medications were classified into therapeutic categories, according to their principle active agent(s), using the *Anatomical-Therapeutic-Chemical Classification System* (ATC) as a reference [22].

Medications that had more than one principle active agent were placed in the therapeutic category of the main principle agent; products with different pharmacological actions were classified taking into consideration the condition for which they were prescribed or used, as described by Coelho Filho, Marcopito, and Castelo [23].

### Ethics

This study was performed in compliance with the Helsinki Declaration and was approved by the Research Ethics Committee of UNESP- the State University of São Paulo (Universidade Estadual Paulista).

### Variables studied

#### Dependent variable

The dependent variable was the use of at least one inappropriate medication by the elderly, defined as the daily use of medications that have been described by the recently updated Beers criteria [16].

With updates to the Beers Criteria, certain medications from the previous list were excluded [18] because no studies based on plausible evidence showed them to be potential causes of adverse reactions in the elderly. On the other hand, other drugs were included and presented as inappropriate medications for use in the elderly, based on the evidence of new studies or because they were recently launched in the market.

Thus, medications inappropriate for the elderly found by the study were classified as “*medications the elderly should avoid, independent of their diseases and conditions*”, “*medications inappropriate for the elderly with certain diseases or syndromes*”, and “*medications that should be used with caution*”, as proposed by the AGS [16]. 

To classify medications as “*medications inappropriate for the elderly with certain diseases or syndromes*”, we examined the records of each senior citizen and the conditions found, duly identified according to the International Classification of Diseases, Version 10 (ICD-10) [24].

We should emphasize that not all of the medications on the Beers list are available in Brazil. Therefore, we evaluated and classified only those medications sold in the country, which were used by the institutionalized elderly participants and included in the updated list.

### Independent variables

Sociodemographic variables and the variables related to health obtained were categorized for the purposes of statistical analysis. Age was categorized as: ≥ 75 and < 75 and hospital admissions as: yes/no, depending on whether the person had or had not been hospitalized during the year prior to the research;

We identified polymorbidity when a patient had four or more diseases [25], and categorized it as follows: no (0–3 diseases) and yes (≥4 diseases).

We measured the dependency level of the elderly using the scale designed by Katz et al. [21] as follows: “dependent” (elderly classified as “dependent” or “partially dependent” by the scale) and “non-dependent” (elderly classified as “independent” by the scale).

We determined polypharmacy by counting the number of medications in use, with a cutoff point of ≥5 medications, as other authors have done [26-28].

An elderly person with a diagnosis of schizophrenia, oligophrenia, or Alzheimer’s disease was considered to have a psychiatric disorder. Cerebrovascular diseases included: epilepsy, CVA sequlae, paraplegia, and atrophy of the limbs.

Elderly people were classified as having depression when the institution’s medical team had made this diagnosis and when they were receiving treatment for this pathology.

### Statistical analysis

Univariate analyses were performed to describe the categorical variables and factors associated with the use of medications inappropriate for the elderly. P-values were generated using the Chi-square association test and Fisher’s exact test.

Next, we performed a multivariate logistic regression model, including in the model any variables with values lower than or equal to 20% in the univariate analysis, which were dependency, polypharmacy, polymorbidity, depression, cerebrovascular disease, psychiatric disorders, and diabetes mellitus.

Through this analysis, we obtained the values for the *odds ratio* (OR), and the significance level adopted was 5%.

All analyses were conducted using Biostat statistical software (version 5.3; Mamirauá Institute for Sustainable Development) [29].

## Results

Out of a total of 268 elderly identified, 3 did not fully reside at the institution (they were there only during the day and returned to their homes at night), and 4 would not yet be 60 years of age within the study period; the remaining 261 were enrolled as participants. Additional file 1: Table S1 shows the characteristics of the participants in this study. Of these, (96.9%) used medication continuously, with an average of 5.7 medications used per day.

**Figure 1:**
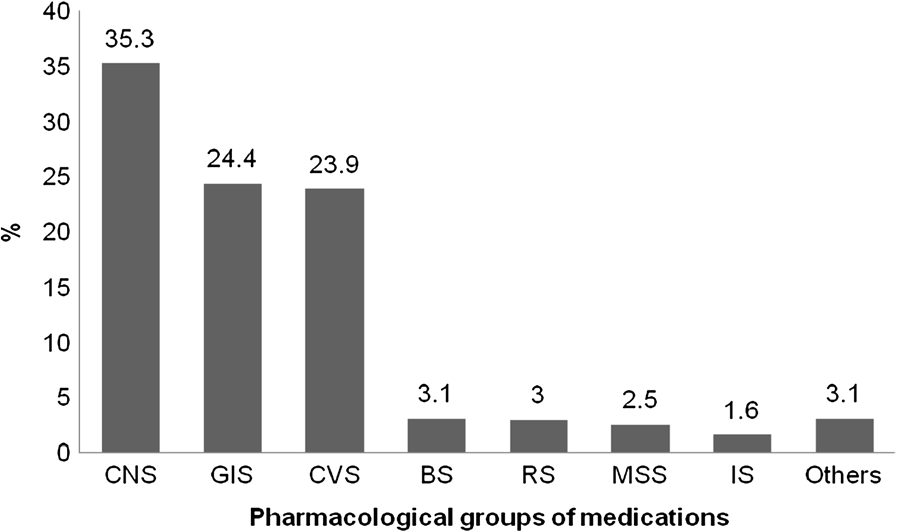
**Pharmacological groups of medications prescribed to residents of care facilities, classified according to the location of action.** CNS = central nervous system; GIS = gastrointestinal system; CVS = cardiovascular system; BS = blood system; RS = respiratory system; MSS = musculoskeletal system; IS = immune system.

The 1452 medications prescribed to this study’s elderly involved 215 different active principles, the majority of which (35.3%) affected the central nervous system (CNS). Figure 1 shows the medications the participants used most, classified into their pharmacological/therapeutic subgroups.

In terms of the inappropriate use of medication, 82.6% of the elderly who used medication on a daily basis received a minimum of one medication that was a PIM, with antipsychotics (26.5%) and analgesics (15.1%) being the most frequently used therapeutic categories (Additional file 2: Table S2).

Anxiolytics (12.5%), antiarrythmics (7.2%), and antidepressants (6.8%) were found in more than 30 prescriptions (Additional file 2: Table S2).

Out of all the medications prescribed and used, 32.4% were PIMs for the elderly. This percentage was divided into the three categories proposed by the AGS as follows: 29.7% PIMs for use by the elderly, independent of any diagnosis; 1.1% medications that were counter-indicated for the elderly with certain pathologies or syndromes; and 1.6% medications that should be used with caution by the elderly (Additional file 2: Table S2).

To analyze associations, we considered the use of at least one medication inappropriate for the elderly. Our univariate analysis revealed a significant association between PIM use and polypharmacy (p ≤ 0.0001), polymorbidity (p ≤ 0.0001), psychiatric disorders (p ≤ 0.0001), depression (p = 0.0204), cerebrovascular disease (p = 0.0048), diabetes mellitus (p = 0.0108) and dependency (p=0.1737) (Additional file 3: Table S3).

Applying multivariate analysis, we observed that the variables of polypharmacy (p = 0.0187), psychiatric disorders (p ≤ 0.0001), cerebrovascular disease (p = 0.0036) and dependency (p=0.0404) were associated significantly with reports of PIM use. However, the variables of polymorbidity, depression and diabetes mellitus lost their significance at this stage of analysis, as the data in Additional file 3: Table S3 shows.

## Discussion

The main results of this study demonstrate a high prevalence for PIM use among elderly Brazilians in institutions, with a predominance of antipsychotics among the therapeutic classes of medications used. We found an association with polypharmacy, a factor consistently reported in the literature. However, certain specific conditions such as psychiatric disorders, dependency, and cerebrovascular diseases were associated factors that we identified for the first time.

### Use of medications

The average consumption of medications used daily by the present study, although it may look high, is actually lower than that of other studies [30-33]. However, in terms of the prevalence of medicines identified as inappropriate, the result observed was potentially high (82.6%).

In terms of the most commonly used therapeutic classes, we observed a predominance of medications acting on the central nervous system (35.3%), with antipsychotics (26.5%) and analgesics (15.1%) being the PIMs most frequently used. These findings corroborate the results of Mann et al. [34] and Fortselund et al. [35]. The predominant use of antipsychotics among the residents of LTCs reflects the high number of older adults affected by mental or behavioral disorders, as reported by Converso and Iartelli [36] and Lenardt et al. [37]. It is to be expected that these disorders are among the main pathologies of residents of LTCs because these elderly people require more care and more time dedicated to them. Therefore, family members end up placing them in LTCs.

### Factors associated with PIM use

We observed a clear association between PIM use and polypharmacy, but not with age, sex, or hospital admissions. Wahruch et al. [25] and Ruggiero et al. [38] reported the same findings. Moreover, numerous studies in the literature [1,32,38-41] have demonstrated a significant relationship between polypharmacy and PIM use. As Bao et al. [41] noted, it is not surprising that polypharmacy is a factor strongly associated with PIM use since patients taking many medications have a better chance of receiving an inappropriate prescription and are more likely to have multiple pathologies, hospitalizations, and consultations with multiple doctors of various specialties, which could lead to prescriptions of PIMs.

Elderly people with cerebrovascular disease and psychiatric disorders have 7.3 and 5.3 times higher chances of using PIMs, respectively, than those unaffected by these illnesses.

According to Wawruch et al. [25], the association between PIM use and certain pathologies could be an important marker of incorrect disease management and a tendency for irrational prescriptions. This finding shows the importance of understanding what conditions or factors are associated with using inappropriate medication, as having this knowledge makes it possible to evaluate the quality of the health care provided.

Another factor associated with PIM use, according to this study’s results, was dependency. Dependent elderly people do not practice activities, not simply physical activities, but also basic and routine activities, which can affect their quality of life; this fact may explain this association.

As a result, this situation can lead to pathologies, such as circulatory disturbances. In addition to these diseases, we verified that polymorbidity occurs in these cases, which in turn leads to a greater consumption of medication and increases the likelihood of receiving an inappropriate prescription, as noted above.

### Strengths of the study and implications for the Brazilian health system

The results of this study are relevant, above all, because there is a dearth of knowledge about pharmacotherapy for elderly Brazilians living in institutions. In addition to addressing a theme about which little is known in Brazil, this study is the first to examine the factors that might influence PIM use in Brazilian LTCs.

We also want to emphasize that this study is one of the first studies to evaluate the use of medications inappropriate for the elderly using the revised Beers criteria [16], and to identify new risk factors for the use of PIMs.

The Beers Criteria list is an indispensible tool to verify the use of inappropriate medications by the elderly and to prevent side effects caused by using inappropriate medications. As a consequence, the use of this tool would reduce the expense of public funds used to finance the health care service (SUS).

Moreover, the results of this study indicate the need to train health professionals responsible for caring for elderly Brazilians living in institutions, given the high prevalence of PIM use.

This result may offer a possible explanation for why Brazilian hospitals and emergency institutions are so overcrowded, given that the PIMs used by 82.6% of the elderly in this study have more risks than benefits and are associated with serious side effects, which are responsible for 10 to 20% of acute hospital admissions [42].

In addition, these results can help administrators and health professionals at these institutions to evaluate their health services, especially pharmacotherapy. Based on these data, it is possible to plan measures to improve medical prescribing in LTCs.

### Limitations of the study

One of the limitations of this study is the fact that the results cannot be generalized to other countries. Moreover, the results are not representative of the elderly living in LTCs in other parts of Brazil, especially the poorest regions. Thus, it is fundamental that additional studies be performed to determine not only the prevalence of inappropriate prescribing, but also its impact on morbimortality within the elderly, especially those in institutions.

Another limitation of this study arises from the fact that the updated Beers list was only recently published. As a result, health professionals may not have had sufficient time to adapt to new scientific evidence. Thus, we need to perform further studies with the same methodological design to verify whether there have been improvements in prescribing appropriate medications to the elderly.

Moreover, we suggest that there should be studies that adapt the updated Beers Criteria to medication available in Brazil. Using the previous Beers-Fick [13] Criteria list, Gorzoni et al. [14] verified that the criteria did not cover all situations involving PIM use in elderly Brazilians. These criteria do not include drugs in common use in Brazil, such as antitussives, cinnarizine, diltiazem, piracetam, quinolones, xanthines, creams, pomades, and eyewashes that should be prescribed with caution to the elderly. As an example, we cite the pharmacological action of certain eyewashes, which may lead to cardiovascular changes and psychiatric disorders in elderly patients [43,44]. Thus, the prevalence of PIM use in this study, although extremely high, may still be underreported.

## Conclusions

This study’s results indicated that there is a high prevalence of PIM use among elderly Brazilians living in institutions, with medications acting on the CNS being the most common group of drugs in use, a circumstance which fits with the high percentage of elderly people who have mental health problems.

We also observed that polypharmacy was a main factor associated with PIM use in residents of institutions, a finding which reflects the multiple pathologies present, and the prevalence of psychiatric disorders, cerebrovascular disease, and dependency.

## Competing interests

The authors declare that they have no conflicts of interests.

## Authors’ contributions

TJVL and CASG were responsible for study concept and design, writing and reviewing of the manuscript. AJIG and DHS carried acquisition of data, data analysis and interpretation, OS performed statistical analysis and critical review of manuscript. All authors read and approved the final manuscript.

## Pre-publication history

The pre-publication history for this paper can be accessed here:

http://www.biomedcentral.com/1471-2318/13/52/prepub

## Supplementary Material

Additional file 1: Table S1 Description of the subjects (N = 261). State of São Paulo, Brazil, 2012. Click here for file

Additional file 2: Table S2 Prevalence of PIMs among the elderly according to the revised Beers criteria, classified according to system of action and therapeutic category. State of São Paulo, Brazil, 2012. Click here for file

Additional file 3: Table S3 Univariate and multivariate analysis of factors associated with PIM use. State of São Paulo, Brazil, 2012. Click here for file
