# Supplementary material for: Potentially inappropriate medications used by the elderly: prevalence and risk factors in Brazilian care homes
Source: BMC Geriatr. 2013 May 30;13:52. doi: 10.1186/1471-2318-13-52 (PMC3679980; doi:10.1186/1471-2318-13-52)
Supplement: Additional file 1: Table S1 — Description of the subjects (N = 261). State of São Paulo, Brazil, 2012. [file 1471-2318-13-52-S1.doc]

| **Table I. Description of the subjects (N = 261). State of São Paulo, Brazil, 2012.** | | |
| --- | --- | --- |
| **Characteristic** | **N** | **%** |
| **Age** |  |  |
| 60-74 | 110 | 42.2 |
| 75-84 | 93 | 35.6 |
| ≥ 85 | 58 | 22.2 |
| **Sex** |  |  |
| Male | 111 | 42.5 |
| Female | 150 | 57.5 |
| **Level of dependency*** |  |  |
| Independent | 137 | 52.5 |
| Partially dependent | 63 | 24.1 |
| Dependent | 61 | 23.4 |
| **Number of medical prescriptions/day** |  |  |
| 0 | 8 | 3.1 |
| 1 | 14 | 5.4 |
| 2 to 4 | 47 | 18 |
| ≥ 5 | 192 | 73.5 |
| **Geriatric conditions** |  |  |
| Mental disorder | 156 | 59.8 |
| Depression | 103 | 39.5 |
| Incontinence | 3 | 1.1 |
| Constipation | 8 | 3.1 |
| Falls and/or lack of balance | 6 | 2.3 |
| **Chronic conditions** |  |  |
| Heart failure | 9 | 3.4 |
| Cerebrovascular disease | 54 | 20.7 |
| Chronic obstructive lung disease | 3 | 1.1 |
| Diabetes mellitus | 62 | 23.7 |
| Peripheral arterial disease | 10 | 3.8 |
| **Other diagnoses** | 256 | 98.1 |

*The Katz Index was used to evaluate the dependency level [21].
