# Supplementary material for: Potentially inappropriate medications used by the elderly: prevalence and risk factors in Brazilian care homes
Source: BMC Geriatr. 2013 May 30;13:52. doi: 10.1186/1471-2318-13-52 (PMC3679980; doi:10.1186/1471-2318-13-52)
Supplement: Additional file 2: Table S2 — Prevalence of PIMs among the elderly according to the revised Beers criteria, classified according to system of action and therapeutic category. State of São Paulo, Brazil, 2012. [file 1471-2318-13-52-S2.doc]

| **Table 2. Prevalence of PIMs for the elderly according to the revised Beers criteria, classified according to the system of action and therapeutic category. State of São Paulo, Brazil, 2012.** | | | | | | | | | | | |
| --- | --- | --- | --- | --- | --- | --- | --- | --- | --- | --- | --- |
| **PIMs independent of medical condition** | | | **PIMs in the presence of certain pathologies** | | | | | **PIMs that should be used with caution** | | | |
| **System/therapeutic category** | **N** | **%** |  | **System/therapeutic category/drug(s)** | **Condition** | **N** | **%** | **System/therapeutic category/drug(s)** | **N** |  | **%** |
| **Central nervous system** | **323** | **75** |  | **Central nervous system** |  | **13** | **81.2** | **Central nervous system** | **24** |  | **100** |
| Antipsychotics | 120 | 27.8 |  | ***Antidepressants*** |  | **5** | **31.2** | ***Anti-epileptics***  carbamazepine | **20** |  | **83.3** |
| Analgesics | 71 | 16.5 |  | Nortriptyline | Dementia, mental disorder | 2 | 12.5 | ***Antidepressants*** |  |  |  |
| Anxiolytics | 59 | 13.7 |  | Paroxetine | Dementia , mental disorder | 2 | 12.5 | mirtazapine | **4** |  | **16.7** |
| Antidepressants | 23 | 5.3 |  | Bupropion | Epilepsy | 1 | 6.2 |  |  |  |  |
| Anticholinergics | 33 | 7.6 |  | ***Antipsychotics*** |  | **5** | **31.2** |  |  |  |  |
| Barbiturates | 17 | 4 |  | Chlorpromazine | Syncope or epilepsy | 5 | 31.2 |  |  |  |  |
| **Cardiovascular system** | **49** | **11.4** |  | ***Antihistamines*** |  | **3** | **19** |  |  |  |  |
| Antiarrythmics | 34 | 8 |  | Pseudoephedrine | Insomnia | 1 | 6.2 |  |  |  |  |
| Antihypertensives | 15 | 3.5 |  | Meclizine | Mental disorder | 1 | 6.2 |  |  |  |  |
| **Respiratory system** | **26** | **6** |  | Loratadine | Mental disorder | 1 | 6.2 |  |  |  |  |
| Antihistamines | 26 | 6 |  | **Gastrointestinal system** |  | **2** | **12.5** |  |  |  |  |
| **Endocrine system** | **14** | **3.2** |  | ***Antimuscarinics*** |  | **1** | **6.2** |  |  |  |  |
| Hypoglycemiants | 14 | 3.2 |  | Oxybutynin | Chronic constipation | 1 | 6.2 |  |  |  |  |
| **Gastrointestinal system** | **7** | **1.6** |  | **Antispasmodics** |  | **1** | **6.2** |  |  |  |  |
| Antispasmodics | 7 | 1.6 |  | Loperamide | Delirium, mental disorders | 1 | 6.2 |  |  |  |  |
| **Skeletomuscular system** | **7** | **1.6** |  | **Cardiovascular system** |  | **1** | **6.2** |  |  |  |  |
| Muscle relaxants | 7 | 1.6 |  | ***Antihypertensives*** |  | **1** | **6.2** |  |  |  |  |
| **Blood system** | **4** | **1** |  | Diltiazem | heart failure | 1 | 6.2 |  |  |  |  |
| Antithrombotics | 4 | 1 |  |  |  |  |  |  |  |  |  |
| **Immune system** | **1** | **0.2** |  |  |  |  |  |  |  |  |  |
| Anti-infection | 1 | 0.2 |  |  |  |  |  |  |  |  |  |
| **Total** | **431** | **100** |  |  |  | **16** | **100** |  | **24** |  | **100** |
