# Supplementary material for: Potentially inappropriate medications used by the elderly: prevalence and risk factors in Brazilian care homes
Source: BMC Geriatr. 2013 May 30;13:52. doi: 10.1186/1471-2318-13-52 (PMC3679980; doi:10.1186/1471-2318-13-52)
Supplement: Additional file 3: Table S3 — Univariate and multivariate analysis of factors associated with PIM use. State of São Paulo, Brazil, 2012. [file 1471-2318-13-52-S3.doc]

| **Table 3. Univariate and Multivariate Analyses of Factors Associated with the Use of Potentially Inappropriate Medications. State of São Paulo, Brazil, 2012.** | | | | | | | | |
| --- | --- | --- | --- | --- | --- | --- | --- | --- |
| ***Variable*** | ***Elderly without PIMs (n = 53 )*** | ***Elderly with PIMs (n = 209)*** | ***p-Value***  ***Chi-Square*** | ***Coefficient*** | ***Standard Deviation*** | ***p-Value Multiple Logistic Regression*** | ***Adjusted OR*** | ***CI 95%*** |
| Age ≥ 75 years | 35 | 116 | 0.2184 | - | - | - | - | - |
| Female sex | 26 | 124 | 0.2321 | - | - | - | - | - |
| Dependency* | 30 | 94 | 0.1737 | -0.7779 | 0.3796 | 0.0404 | 0.4594 | 0.22 to 0.97 |
| Polypharmacy | 25 | 167 | < 0.0001 | 0.9558 | 0.4063 | 0.0187 | 2.6007 | 1.17 to 5.77 |
| Hospital admissions | 7 | 27 | 0.8996 | - | - | - | - | - |
| Polymorbidity | 4 | 80 | < 0.0001 | 1.0911 | 0.6802 | 0.1087 | 2.9776 | 0.79 to 11.29 |
| Psychiatric disorders | 13 | 126 | < 0.0001 | 1.6793 | 0.4025 | <0.0001 | 5.3518 | 2.44 to 11.80 |
| Depression | 10 | 77 | 0.0204 | 0.4693 | 0.4693 | 0.3173 | 1.5988 | 0.64 to 4.01 |
| Cerebrovascular disease | 3 | 51 | 0.0048 | 1.9928 | 0.6842 | 0.0036 | 7.3357 | 1.92 to 28.05 |
| Diabetes mellitus | 5 | 57 | 0.0108 | 0.8552 | 0.5636 | 0.1292 | 2.3519 | 0.78 to 7.10 |

*The scale used to evaluate dependency was the Katz Index [21];

*p*: statistical significance;

*Adjusted OR:* odds ratio or odds ratio adjusted for all the variables;

*CI:* confidence interval.
